# Treatment of Nausea and Vomiting in Pregnancy: Factors Associated with ED Revisits

**DOI:** 10.5811/westjem.2016.6.29847

**Published:** 2016-07-21

**Authors:** Brian R. Sharp, Kristen M. Sharp, Brian Patterson, Suzanne Dooley-Hash

**Affiliations:** * University of Wisconsin School of Medicine and Public Health, BerbeeWalsh Department of Emergency Medicine, Madison, Wisconsin; † University of Wisconsin School of Medicine and Public Health, Department of Obstetrics and Gynecology, Madison, Wisconsin; ‡ University of Michigan Medical School, Department of Emergency Medicine, Ann Arbor, Michigan

## Abstract

**Introduction:**

Nausea and vomiting in pregnancy (NVP) is a condition that commonly affects women in the first trimester of pregnancy. Despite frequently leading to emergency department (ED) visits, little evidence exists to characterize the nature of ED visits or to guide its treatment in the ED. Our objectives were to evaluate the treatment of NVP in the ED and to identify factors that predict return visits to the ED for NVP.

**Methods:**

We conducted a retrospective database analysis using the electronic medical record from a single, large academic hospital. Demographic and treatment variables were collected using a chart review of 113 ED patient visits with a billing diagnosis of “nausea and vomiting in pregnancy” or “hyperemesis gravidarum.” Logistic regression analysis was used with a primary outcome of return visit to the ED for the same diagnoses.

**Results:**

There was wide treatment variability of nausea and vomiting in pregnancy patients in the ED. Of the 113 patient visits, 38 (33.6%) had a return ED visit for NVP. High gravidity (OR 1.31, 95% CI [1.06–1.61]), high parity (OR 1.50 95% CI [1.12–2.00]), and early gestational age (OR 0.74 95% CI [0.60–0.90]) were associated with an increase in return ED visits in univariate logistic regression models, while only early gestational age (OR 0.74 95% CI [0.59–0.91]) was associated with increased return ED visits in a multiple regression model. Admission to the hospital was found to decrease the likelihood of return ED visits (p=0.002).

**Conclusion:**

NVP can be difficult to manage and has a high ED return visit rate. Optimizing care with aggressive, standardized treatment in the ED and upon discharge, particularly if factors predictive of return ED visits are present, may improve quality of care and reduce ED utilization for this condition.

## INTRODUCTION

### Background

Nausea and vomiting in pregnancy (NVP) refers to a spectrum of symptoms that affect 50–90% of all pregnant women, typically in the first trimester and can adversely affect both maternal and fetal health.^1^–^3^ Hyperemesis gravidarum (HEG) represents the most severe form of NVP and is present in approximately 0.5 to 2 percent of pregnancies.^1^,^4^,^5^ HEG is often characterized by maternal weight loss and fluid, electrolyte, and nutritional abnormalities^3^ and is the most common indication for hospitalization during early pregnancy with an average of 1.3 hospital admissions per HEG patient. It is second only to preterm labor as the most common reason for hospitalization during pregnancy^6^,^7^ and carries up to a 25% hospital readmission rate.^8^ HEG is also the most common cause of pregnant patients missing time at work (average hospital stay of 2.6–4 days) and reduced quality of life.^1^,^8^ The obstetric literature suggests factors predisposing patients to the severe variants of NVP include nulliparity, younger patient age, non-white race, and the presence of comorbidities including pre-existing diabetes, depression or other psychiatric illness, asthma, and hyperthyroid disease.^1^,^9^ Although diagnostic tests indicative of starvation and dehydration such as ketonuria, abnormal electrolytes, liver function tests, and hematocrit have traditionally been used as markers for severe NVP, there have been multiple studies showing that these may not successfully predict hospital readmission.^4^,^9^

Patients with NVP and HEG are frequently assessed and treated in the emergency department (ED), yet little is known regarding the quality of care that they receive or their rates of ED utilization. We are aware of no peer-reviewed publications to date in either the ED or obstetric literature that specifically assesses the care of this condition in the ED or provides guidelines for its treatment in this setting. The American College of Obstetrics and Gynecology (ACOG) has published practice guidelines on the treatment of NVP that are often referenced for this condition but are not specifically focused on care in the ED.^10^

We hypothesize that NVP treated in the ED does indeed have a high re-visit rate and that the variables of decreased patient age, decreased gestational age, decreased maternal gravidity, the presence of multiple gestation pregnancies, and the presence of lab abnormalities are associated with a higher likelihood for return ED visits for treatment of NVP. We also aimed to assess treatment patterns of NVP in the ED.

## METHODS

### Study Design and Setting

We conducted a retrospective database analysis the electronic medical record from a single, large academic hospital. Institutional review board (IRB) approval was granted in advance of this study.

### Study Population

Patients who met inclusion criteria were adult women treated in the ED between 1/1/10 and 12/31/10 who received ICD-9 diagnosis codes for variants of “nausea and vomiting in pregnancy” or “hyperemesis gravidarum.”

### Measures

A manual chart review by a single reviewer was conducted on identified patients with demographic and treatment variables collected via standardized collection form (Appendix 1 and 2).

Demographic and historic variables included age, gravity, parity, gestational age, comorbidities, and multiple gestations. Treatment variables abstracted included type, amount, and timing of antiemetics administered, type and amount of IV fluids given, length of ED stay, disposition, and discharge prescriptions. Our primary outcome was return visit to the ED at any point during gestation for NVP.

The methods of this study did follow published methodological criteria for a medical record study for abstractor training, case selection criteria, variable definition, abstractor forms, performance monitoring, medical record identification, sampling method, and IRB approval. The abstractor was not blinded to the hypothesis, there was no inter-observer reliability measured (due to a single reviewer), and there were no missing data.^11^

### Data Analysis

Descriptive statistics were used to evaluate treatment trends. We used logistic regression analysis to examine the relationships between all variables and the primary study outcome of return visits to the ED for NVP. For the purpose of regression modelling, we defined binary indicators for the followng: presence of ketonuria, presence of multiple gestations, obstetrics consultation on initial ED visit, admission, and the administration of Phenergan or odansetron (in any quantity) in the ED. Age, gravity, partiy, gestational age (in weeks), ED length of stay (minutes), and number of liters of total IV fluids given were analyzed as continuous variables. We examined variables individually, and we included those that were significantly associated with readmission in a multiple logistic model. These variables were chosen a priori by author consensus, consistency with cited previous literature, and availability for analysis. We conducted all statistical analyses using STATA (Version 14, College Station, TX).

## RESULTS

In the study year, 62,473 adult patients presented to the study site ED. Of these, 113 patient visits were identified that met inclusion criteria for this study and were found to have a mean age of 27.1 years (SD±5.25), mean gravidity of 2.90 pregnancies (SD±1.94), and mean gestational age of 8.78 weeks (SD±3.21).

The mean overall length of ED evaluation was 730 min (SD±513), which included 49 of the 113 patients (43%) whose treatment included placement in ED observation status. When ED-based observation status patients were removed, mean length of ED stay was 389.18 minutes (SD±228.63). Of the 113 patient visits, 17 (15%) ultimately resulted in hospital admission and 95 (85%) were discharged home either from the ED or ED-based observation status (Figure).

We observed wide variation in treatment approaches to NVP in the ED (Table 1). Seven different antiemetics were used in the ED. Ondansetron was most commonly used—99 patients (87.6%) received ondansetron, 39 patients (34%) promethazine, and 39 patients (34%) prochlorperazine. In total, ondansetron constituted 191/282 (68%) of all total doses of antiemetic administered in the ED. Only 37% of patients received more than one different antiemetic agent while in the ED with a mean maximum time between doses of antiemetics of 359.22 minutes (SD±221.7). Antiemetics were predominantly administered intravenously with 4 of 95 discharged patients (4.2%) receiving oral or rectal antiemetics prior to discharge home. Only 6 of 95 discharged patients (6.4%) received prescriptions for, or were previously on, vitamin B6. Ondansetron was the most common discharge prescription provided (44/99 prescriptions written), while 36 of 95 discharged patients (37.9%) received prescriptions for anything other than, or in addition to, ondansetron.

There were five different types of IV fluids administered with a mean number of liters received of 2.96 (SD±1.64). Of the total 333 liters of IV fluids given, the most commonly administered was normal saline (188 liters; 56%), followed by D5LR (78 liters; 23.4%) and D5.45 (54 liters; 16%). In total, 77% of patients (87) received normal saline; 23% D5.45 (26) and 21.3% D5LR (24).

High gravidity, high parity and early gestational age were associated with an increase in return ED visits in a univariate logistic regression model, while only early gestational age (OR 0.74 95% CI [0.59–0.91]) were associated with an increase of return ED visits in a multivariate model.

Of the 113 ED patient visits, 38 (33.6%) had a return ED visit for NVP with a total of 25/77 (32.5%) of individual patients representing more than one of the ED visits. None of the 17 patients admitted on their initial ED visit returned (p=0.002) in a two-tailed test of proportion; as a perfect predictor this variable was not to be included in the univariate or multiple regression models. In univariate regression analysis, only three of our putative predictor variable demonstrated significant association with readmission: Increasing gestational age was associated with reduced risk of readmission (OR 0.74 95% CI [0.60–0.90]), while increasing parity (OR 1.50 95% CI [1.12–2.00]) and gravidity (OR 1.31 95% CI [1.06–1.61]) were associated with increased risk of revisits. In multiple regression analysis including only these three variables, only the association with increasing gestational age remained significant (OR 0.74 95% CI [0.60–0.90]). No other variables were found to be predictive of return visits to the ED including those classically associated with severe forms of nausea and vomiting in pregnancy including the presence of ketonuria, a multiple gestation pregnancy, or electrolyte abnormalities. No treatments (type of antiemetic or IV fluid) had a statistically significant impact on either admission rate or rate of return visits to the ED (Table 2).

## DISCUSSION

As a paucity of literature exists to provide best practices for treatment of NVP specifically in the ED setting, practitioners often rely on established practice patterns for treating nausea and vomiting of other etiologies. Comparing our results to current ACOG guidelines for general treatment of NVP, several trends emerge that may represent potential opportunities for treatment optimization in the ED and upon discharge home.

In this study, antiemetics administered in the ED largely consisted of monotherapy with parental ondansetron. Ondansetron is often used as the antiemetic of choice for patients presenting to the ED with various conditions causing nausea and vomiting due to its efficacy and favorable side effect profile. Although the ACOG guidelines do recommend metoclopramide (Category B) and promethazine (Category C) in their treatment algorithm^10^ before ondansetron (Category B), considering ED provider familiarity, it is not an unreasonable starting point for treatment of NVP in the ED. However, a more aggressive and earlier initiation of multi-agent antiemetic therapy should be considered either in place of, or in addition to ondansetron (despite the increased risk of extrapyramidal effects from these other medications) when considering the long average ED length of stay in this study with patients primarily receiving monotherapy with ondansetron. This may have more relevance with the increasingly controversial safety profile of ondansetron use in pregnancy with recent evidence suggestive of possible increases in fetal complications including cardiac malformations, development of cleft palate, and other major congenital malformations.^12^–^15^

Although patients presenting to the ED with various conditions causing nausea and vomiting are frequently treated primarily with parental therapy, given the frequent refractory nature of NVP, it may be reasonable to also consider not only a trial of oral intake but also attempting symptom control with either oral or rectal antiemetic agents to ensure successful outpatient symptom control. This becomes more compelling when taking into account the already long mean ED length of stay observed in this study, one that often includes observation status care—potentially indicating the ability to accommodate a trial of alternate route of antiemetic therapy without significantly impacting ED length of stay or utilization while possibly reducing recurrent ED visits.

The majority of patients in this study were discharged home without any prescriptions for new or additional antiemetics. Many patients were already on ondansetron at home and this was the most commonly prescribed in our study. Very few patients were discharged with prescriptions for other antiemetic therapy recommended by ACOG including metoclopramide or promethazine, and very few received prescriptions for the other ACOG first line recommended agents, pyridoxine and doxylamine. Given the recurrent nature of NVP, inadequate treatment of recurrent symptoms after discharge may have contributed to the frequent return ED visits seen in these patients. Although impossible to attribute causality to the optimization of discharge medications as preventing repeat ED visits, it is interesting to further consider that none of the 17 patients ultimately admitted to the inpatient obstetric service had a repeat presentation to the ED. Improved discharge planning and care could potentially contribute to reducing recurrent ED visits for this condition.

A significant portion of our patients’ ED visits (43%) included extended observation care status. Although observation level care did not significantly reduce rate of ED re-presentation, NVP does seem to be an ideal condition for ED observation care with the opportunity for extended treatment and optimization of treatment. This could include a trial of oral challenge and symptom control with non-parental antiemetics. This also provides a potential opportunity to ensure necessary outpatient resources such as adequate prompt follow up or even OB involvement while in the ED—particularly if symptoms prove refractory or if the patient is presenting recurrently to the ED.

This study demonstrated a high overall rate of return ED visits. Identifying factors associated with recurrent ED visits such as lower gestational age, or high gravidity and parity, may potentially allow care providers to more effectively target increasing attention towards care optimization for high-risk patients using the methods discussed above.

In order to optimize treatment of NVP in the ED, it appears that we may need to shift our treatment approach. This should include consideration of replacement of or supplementation of ondansetron monotherapy with a more diverse and aggressive treatment strategy involving multiple antiemetics, more extensive outpatient prescriptions in accordance with ACOG’s recommendations, and ensuring close obstetric follow up with a potential role for extended observation status or an observation unit stay. We are in the process of developing a more standardized protocol for treatment of NVP with plans to study similar outcomes after treatment is more clearly protocolized with regards to antiemetic agent selection, route and frequency of anti-emetics, and prescriptions for home anti-emetics.

## LIMITATIONS

Some limitations are inherent to this study and its design that impact its generalizability. The sample size in this study is small but is consistent with the other studies of NVP and HEG published in the obstetric literature. The fact that this study was conducted at a single site could have implications if certain treatment patterns are more prevalent on an institutional or regional level and may not necessarily be representative of wider ED practice patterns.

Patients were identified for inclusion in this study if they received an ICD-9 diagnosis code of variants of “nausea and vomiting in pregnancy” or “hyperemesis gravidarum.” It is possible that patients presented with this disease process but received alternative or less specific diagnostic coding (i.e gastritis, nausea, or vomiting) and were missed from study inclusion. Because we only identified patients who came through the ED, this creates the possibility of missing patients who were admitted via alternative mechanisms such as direct admission. However, we were able to see if any of the patients initially meeting criteria by coming through the ED were subsequently directly admitted to the hospital.

Lastly, the retrospective study design limited our ability to gather any additional information from the patient that may have been helpful if conducted prospectively including quantitative beta-hCG levels (which are not routinely obtained in treatment of these patients but could have potential to predict severe variants of NVP), compliance with medications, dietary patterns, or other demographic variables not already contained in the EMR such as living situation, or presence of other stressors. This also limited our ability to assess follow-up information on patients with out-of-network providers such as visits to other hospitals or EDs, and subsequent obstetric follow-up care and outcomes.

## CONCLUSION

Despite being a condition commonly seen in the emergency department, there is limited literature focused on specifically assessing or guiding treatment of NVP in the ED. NVP can be refractory to therapies commonly employed for other cause of nausea and vomiting, and in this study demonstrated a high ED return visit rate. To our knowledge, this study represents the first systematic attempt to identify patient and treatment factors that predict which patients are at highest risk of return visits. The wide variance in practice patterns combined with high return rate point to an opportunity for improving quality and consistency of care. Further work is needed to develop treatment guidelines for ED-specific care of NVP as well as improve discharge and follow-up planning to help reduce ED utilization. With the increasing pressure on healthcare systems to reduce hospital admission and, in particular, readmissions, this type of information will become increasingly important in the ED setting.

## Supplementary Information





## Figures and Tables

**Figure:**
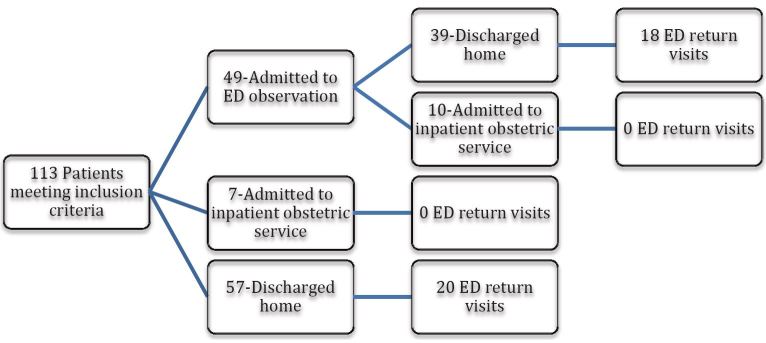
Study flow diagram. *ED,* emergency department

**Table 1:** Means and standard deviations of demographic and treatment variables.

Variable	Mean	SD
Patient age	27.1	5.25
Gestational age (weeks)	8.78	3.21
Gravity	2.9	1.94
Parity	1.38	1.38
ED LOS (minutes)	730	513
Total doses of antiemetics	2.5	2.01
Number of types of antiemetics	1.35	0.693
IV fluid volume (L)	2.96	1.64

*ED,* emergency department; *LOS,* length of stay; *IV,* intravenous

**Table 2:** Summary of logistic regression analysis for the primary outcome.

Variable	Univariate odds ratio (95% CI)	Multiple odds ratio (95% CI)
Age (years)	1.07(0.99–1.16)	N/A
Gravity	1.31(1.06–1.61)	0.81(0.45–1.46)
Parity	1.50 (1.12–2.00)	1.86(0.82–4.24)
Gestational age (weeks)	0.74 (0.60–0.90)	0.74 (0.59–0.91)
Multiple gestation	2.06 (0.39–10.77)	N/A
Electrolyte abnormality	0.36 (0.07–1.74)	N/A
Admission	No admitted patients returned to the hospital	
Ketonuria	0.89 (0.36–2.22)	N/A
Anti-emetics prescribed on discharge	1.90(0.83–4.31)	N/A
Obstetrics-gynecology consulted	0.82 (0.29–2.33)	N/A
Number of liters of IVF	1.14(0.90–1.45)	N/A
ED evaluation in minutes	1.00 (0.99–1.00)	N/A
Ondansetron given in ED	1.46(0.43–4.94)	N/A
Promethazine given in ED	0.87(0.39–1.98)	N/A

*ED,* emergency department, *IVF,* intravenous fluids

## References

[1] Bailit JL (2005). Hyperemesis gravidarum: Epidemiologic findings from a large cohort. Am J Obstet Gynecol.

[2] Jewell D, Young G (2003). Interventions for nausea and vomiting in early pregnancy. Cochrane Database Syst Rev.

[3] Niebyl JR (2010). Nausea and Vomiting in Pregnancy. N Engl J Med.

[4] Eliakim R, Abulafia O, Sherer DM (2000). Hyperemesis gravidarum: a current review. Am J Perinatol.

[5] Gadsby R, Barnie-Adshead AM, Jagger C (1997). Pregnancy nausea related to women’s obstetric and personal histories. Gynecol Obstet Invest.

[6] Adams MM, Harlass FE, Sarno AP (1994). Antenatal hospitalization among enlisted service-women, 1987–1990. Obstet Gynecol.

[7] Gazmararian JA, Petersen R, Jamieson DJ (2002). Hospitalizaitons during pregnancy among managed care enrollees. Obstet Gynecol.

[8] Tan PC, Omar SZ (2011). Contemporary approaches to hyperemesis during pregnancy. Curr Opin Obstet Gynecol.

[9] Fell DB, Dodds L, Joseph KS (2006). Risk factors for hyperemesis gravidarum requiring hospital admission during pregnancy. Obstet Gynecol.

[10] American College of Obstetrics and Gynecology ACOG (American College of Obstetrics and Gynecology) (2004). Practice Bulletin: nausea and vomiting of pregnancy. Obstet Gynecol.

[11] Worster A, Bledsoe D, Cleve P (2005). Reassessing the methods of medical record review studies in emergency medicine. Ann Emerg Med.

[12] Koren G (2014). Treating morning sickness in the United States—changes in prescribing are needed. Am J Obstet Gynecol.

[13] Einarson A, Maltepe C, Navioz Y (2004). The safety of ondansetron for nausea and vomiting of pregnancy: a prospective comparative study. BJOG.

[14] Pasternak B, Svanström H, Hviid A (2013). Ondansetron in pregnancy and risk of adverse fetal outcomes. N Engl J Med.

[15] Anderka M, Mitchell AA, Louik C, National Birth Defects Prevention Study (2012). Medications used to treat nausea and vomiting of pregnancy and the risk of selected birth defects. Birth Defects Res A Clin Mol Teratol.

