# Supplementary material for: Treatment of Nausea and Vomiting in Pregnancy: Factors Associated with ED Revisits
Source: West J Emerg Med. 2016 Jul 21;17(5):585–90. doi: 10.5811/westjem.2016.6.29847 (PMC5017843; doi:10.5811/westjem.2016.6.29847)
Supplement: Supplementary file 1 [file wjem-17-585-s001.pdf]

Patient Study ID____________

Date of Service____________

Age_______y/o Gravity_______ Parity________ Gestational Age_______weeks

Multiple Gestation (yes/no) Prior Pregnancy Complications (yes/no)

Medical Problems Listed in PMH _____________________________ __

Listed or noted home antiemetics prior to ED presentation (yes/no)

-If yes, which type(s)____

ED Length of Stay_______minutes
-Placed in ED Observation Protocol/status (yes/no)
-Admitted (yes/no)
-Recurrent ED? (yes/no)
-OB/GYN Consulted (yes/no)

Electrolyte abnormalities during ED encounter outside of institutional lab parameters (yes/no)

-If yes, what lab values____

Ketonuria present (yes/no)

-If yes, amount of initial ketonuria_________________mg/dl
