# Supplementary material for: Treatment of Nausea and Vomiting in Pregnancy: Factors Associated with ED Revisits
Source: West J Emerg Med. 2016 Jul 21;17(5):585–90. doi: 10.5811/westjem.2016.6.29847 (PMC5017843; doi:10.5811/westjem.2016.6.29847)
Supplement: Supplementary file 2 [file wjem-17-585-s002.pdf]

ED Treatment Variables:

Antiemetic medications administered while the patient was in the ED:

Dose 1: Type______________ Dose/Route______________ Time Administered____________

Dose 2: Type______________ Dose/Route______________ Time Administered____________

Dose 3: Type______________ Dose/Route______________ Time Administered____________

Dose 4: Type______________ Dose/Route______________ Time Administered____________

Dose 5: Type______________ Dose/Route______________ Time Administered____________

Dose 6: Type______________ Dose/Route______________ Time Administered____________

Dose 7: Type______________ Dose/Route______________ Time Administered____________

Dose 8: Type______________ Dose/Route______________ Time Administered____________

IV Fluid Therapy administered while the patient was in the ED:

IV Fluid 1: Type______________ Volume______________mL Time Administered____________

IV Fluid 2: Type______________ Volume______________mL Time Administered____________

IV Fluid 3: Type______________ Volume______________mL Time Administered____________

IV Fluid 4: Type______________ Volume______________mL Time Administered____________

IV Fluid 5: Type______________ Volume______________mL Time Administered____________

IV Fluid 6: Type______________ Volume______________mL Time Administered____________

Antiemetic prescriptions provided upon discharge from the ED:

Medication 1: Type______________ Dose/Route______________

Medication 2: Type______________ Dose/Route______________

Medication 3: Type______________ Dose/Route______________

Medication 4: Type______________ Dose/Route______________

Other Relevant Notes:
